# TD-DFT Evaluation of Electronic Spectra of Coumarin and Anthraquinone Dyes: Effects of Functionals, Basis Sets, and Solvation Conditions

**DOI:** 10.1021/acsomega.5c10203

**Published:** 2026-01-17

**Authors:** Guilherme Gustavo Silva Amorim, Paula Homem-de-Mello, Rogério Custodio

**Affiliations:** † Instituto de Química, 344102 Universidade Estadual de Campinas, P.O. Box 6154, Barão Geraldo, Campinas, São Paulo 13083-970, Brazil; ‡ Centro de Ciências Naturais e Humanas (CCNH), 74362 Universidade Federal do ABC (UFABC), Avenida dos Estados 5001, Santo André, São Paulo 09210-580, Brazil

## Abstract

The electronic absorption spectra of 20 organic dyes (7 coumarins, 10 anthraquinones, and 3 other similar molecules) were investigated using TD-DFT with BLYP, B3LYP, and B3PW91 functionals and four basis sets: 6-31++G­(d,p), 6-311++G­(2df,p), aug-cc-pVDZ, and aug-cc-pVTZ. Gas-phase, implicit, and hybrid solvation models were tested. The hybrid model, guided by electrostatic potential maps, yielded the lowest mean absolute errors with respect to experimental absorption maxima, underscoring the role of specific electrostatic solute–solvent interactions. Overall, the solvent effects were most pronounced with B3LYP and B3PW91. Replacing the LYP correlation functional with PW91 led to shifts of up to 6 nm, while modifying the exchange term from B to B3 caused deviations of at least 33 nm. Basis set performance was consistent across molecules, showing approximately linear trends. Within this protocol, electrostatic-potential-guided microsolvation provides an efficient way to capture key solute–solvent interactions without resorting to more demanding QM/MM approaches.

## Graphical abstract

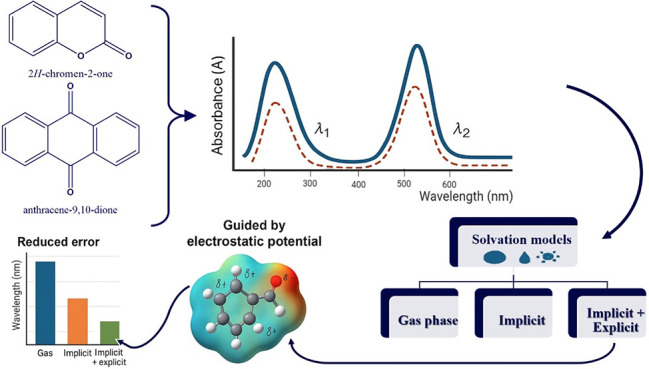

## 1 Introduction

Organic dyes play a central role in numerous scientific and technological applications, primarily due to their light absorption and emission capabilities.^1–9^ Among these compounds, anthraquinones and coumarins stand out not only for their optical properties but also for their broad range of biological activities, including anticancer, antioxidant, antibacterial, antiviral, antimalarial, antidiabetic, and antifungal effects.^10–13^

For anthraquinones, theoretical investigations using time-dependent density functional theory (TD-DFT) have been employed to explore their photochromic behavior, HOMO–LUMO energy gaps, and the influence of conjugation, solvent environment, and excitation energy on their spectroscopic features.^14,15^ Additional TD-DFT studies have examined substituent effects and solvent-induced shifts in anthraquinone derivatives, further expanding the understanding of their electronic structure.^16–21^ These molecules have also been studied as potential pharmacological agents, with particular attention to how their electronic properties (such as chemical stability, polarizability, and charge-transfer characteristics) relate to their biological activity.^22,23^

In the case of coumarins, TD-DFT calculations have shown excellent agreement with experimental absorption and fluorescence spectra, underscoring the significance of solvation effects and validating the applicability of Kasha’s rule in these systems.^24^ Beyond vertical excitation energies, some studies have modeled complete absorption and emission spectra for selected coumarin derivatives, combining TD-DFT with explicit/implicit solvation and vibronic structure, as in the work of Cerón-Carrasco and coworkers on 7-hydroxycoumarin.^25^ Several groups have reported systematic TD-DFT benchmarks for coumarin derivatives, evaluating functional performance and substituent effects across different spectral regions.^26–31^ Comparative analyses using different functionals, including B3LYP and CAM-B3LYP, have highlighted the importance of selecting appropriate computational methods to accurately predict absorption maxima. These studies have also emphasized the impact of electron-donating and electron-withdrawing substituents on the electronic structure of coumarins.^30,32–41^ Collectively, these findings reinforce the utility of TD-DFT for the theoretical characterization of organic dyes, enabling reliable predictions of their optical and electronic behavior.^42^

A major strength of TD-DFT lies in its balance between computational efficiency and predictive accuracy, especially when compared to more computationally demanding correlated methods such as Coupled Cluster or Full Configuration Interaction.^43^ Its flexibility in the choice of exchange–correlation functionals allows the method to be adapted to the characteristics of specific molecular systems.^44,45^ However, this same versatility poses a challenge: the large number of available functional–basis set combinations can complicate the identification of optimal protocols, necessitating careful methodological calibration.

An equally critical factor in simulating the optical properties of dyes is the environment in which they are embedded. Solvent effects, for instance, can cause substantial shifts in excitation wavelengths and transition intensities.^45,46^ Consequently, for systems studied in solution, typical in most experimental systems, accurate theoretical descriptions of the spectra require models that closely reproduce the experimental environment.^47^ Various strategies have been proposed to incorporate solvent effects, including implicit solvation models,^48–50^ explicit solvent approaches, and hybrid methods that combine both representations.^25,51^ Several studies have provided in-depth evaluations of these methodologies.^52,53^ However, hybrid solvation models raise practical considerations, such as determining the appropriate number and placement of explicit solvent molecules. One underexplored strategy to guide this placement involves the use of electrostatic potential (ESP) maps to identify regions of high electron density and likely solvent interaction sites.^54^ In this context, it becomes relevant to assess, in a systematic way, how different solvation choices (implicit, explicit, and ESP-guided hybrid) affect the description of electronic transitions.

Given the relevance of anthraquinone- and coumarin-based dyes and the pronounced influence of solvation on their electronic properties, the present work aims to investigate the electronic absorption spectra of these dye classes using TD-DFT under distinct solvation conditions. Specifically, the influence of the choice of exchange–correlation functional, basis set, and solvation model, including gas-phase, implicit, and ESP-guided hybrid representations, on the computed electronic transitions is assessed in order to elucidate the extent to which these variables affect the optical response of such systems. To this end, a consistent protocol is adopted in which three widely used functionals (B3LYP, B3PW91, and BLYP) and four basis sets are applied to a set of 20 dyes. This design makes it possible to disentangle the effects of exchange and correlation by selecting functionals that share either the same exchange or the same correlation component and enables a comparison of solvation models under tightly controlled conditions. In this first step, our analysis is deliberately restricted to vertical absorption maxima (λ_max_) and associated error metrics, rather than to full spectral profiles, so as to compare in a controlled way the effect of functional, basis set, and solvation model across a broader set of dyes.

## 2 Computational Methods

Electronic absorption spectra were computed using time-dependent density functional theory (TD-DFT).^55^ To investigate the influence of exchange and correlation effects, we selected three representative functionals: B3LYP,^56^ BLYP,^57^ and B3PW91.^58^ The comparison between B3LYP and BLYP isolates the role of exchange, while differences between BLYP and B3PW91 highlight variations in the correlation treatment. For each functional, calculations were carried out using four basis sets of increasing flexibility and accuracy: 6-31++G­(d,p),^59–62^ 6-311++G­(2df,p),^59–62^ aug-cc-pVDZ,^63,64^ and aug-cc-pVTZ.^63,64^ All computations were performed with the *Gaussian09* suite of programs.^65^

In order to avoid missing electronic transitions with appreciable oscillator strength within the UV–vis region of interest, the lowest 60 singlet excited states were computed for each dye, providing adequate spectral coverage for all exchange–correlation functionals and solvation approaches considered. For comparison with experimental data, the selected transition was the one closest to the experimental absorption maximum, giving preference to the state with the highest oscillator strength when multiple candidates were possible. When the experimental absorption was restricted to the visible region, transitions within this range were prioritized, even if the most intense excitation occurred in the UV.

Solvent effects on the spectral properties were explored under three different conditions: 1) gas-phase (vacuum): calculations were performed on isolated molecules without any solvent model; 2) implicit solvation: using the SMD (Solvation Model based on Density) continuum model,^50^ which treats the solvent as a polarizable medium surrounding the solute; and 3) hybrid solvation: combining the implicit SMD model with the addition of explicit water molecules positioned near selected regions of the solute. In this hybrid approach, two configurations were employed:

(a) Water molecules were placed near hydrogen-bonding sites, such as OH and NH groups, and, for the fluorinated dye, near the fluorine atoms of the CF_3_ group or near carboxylic groups in acidic dyes.

(b) One water molecule was placed near each region of high negative electrostatic potential, as identified by electrostatic potential mapping. Consequently, the number of explicit water molecules varied according to the number of negative ESP regions present in each dye.

The accuracy of the theoretical predictions was evaluated using the mean absolute error (MAE), defined as

1 $$EAM=\frac{1}{n}\overset{n}{\underset{i=1}{\sum }}\left|\lambda_{calc}-\lambda_{exp}\right|$$

where λ_calc_ and λ_exp_ denote the computed and experimental absorption maxima, respectively. MAE is widely used in the literature due to its straightforward interpretation and effectiveness in quantifying the deviation between theory and experiment.

## 3 Results and Discussion

Figure 1 presents the 20 molecular structures analyzed, composed mainly of anthraquinone and coumarin dyes, along with three related benzenoid derivatives (H_2_DHBQ, DHBQ^2–^, and BMESPA). The optimized geometries and the selected electronic transitions for all systems, obtained under different computational conditions, are provided as the Supporting Information. Table 1 summarizes the mean absolute errors (MAEs) for the UV–vis spectra calculated using the BLYP, B3LYP, and B3PW91 functionals across various basis sets and solvation models. Errors are reported in nanometers (nm). As shown in Table 1, BLYP yielded the highest MAE values under all solvation conditions, indicating its lower reliability in predicting UV–vis spectra. In contrast, B3LYP and B3PW91 exhibited similar behavior, with significantly lower MAEs compared with BLYP. For both functionals, the largest deviations were observed under gas-phase conditions. The introduction of implicit solvation substantially reduced the errors, while the inclusion of explicit water molecules produced only marginal improvements. This suggests that the specific strategy used for placing water molecules had a limited impact on enhancing agreement with experimental data.

**Figure 1:**
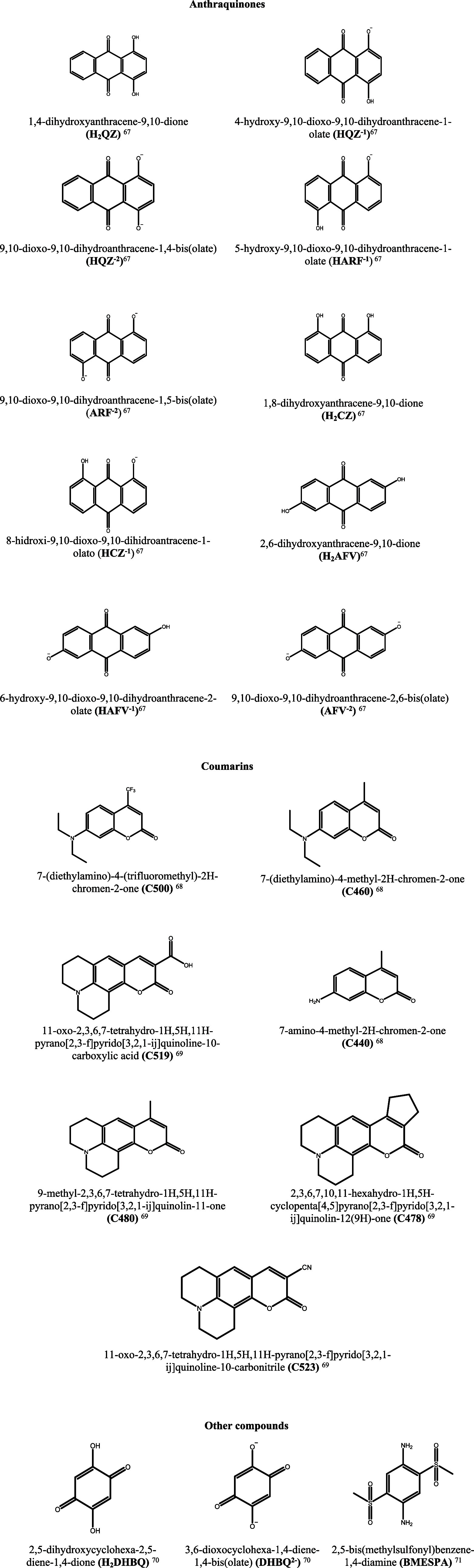
Molecular structures of the dyes studied, accompanied by their respective names and abbreviations.^67–71^ .

**Table 1:** Mean Absolute Errors (MAEs, in nm) of the UV–vis Spectra Calculated with the BLYP, B3LYP, and B3PW91 Functionals, Using Different Basis Sets and Solvation Strategies: Gas Phase, Implicit Solvation, and Implicit Solvation Combined with Explicit Water Molecules Placed Near OH and NH Groups as well as around Fluorine Atoms

Functional	Basis set	Gas phase	Implicit solvent	Implicit + explicit solvent
BLYP	6-31++G(d,p)	55	57	55
	6-311++G(2df,p)	53	56	54
	aug-cc-pVDZ	55	59	57
	aug-cc-pVTZ	53	55	54
B3LYP	6-31++G(d,p)	42	16	17
	6-311++G(2df,p)	41	16	15
	aug-cc-pVDZ	41	18	18
	aug-cc-pVTZ	41	14	15
B3PW91	6-31++G(d,p)	44	17	16
	6-311++G(2df,p)	43	16	14
	aug-cc-pVDZ	44	17	17
	aug-cc-pVTZ	43	14	15

The comparison between B3LYP and B3PW91 indicates that differences between the LYP and PW91 correlation components had little influence on the results. However, replacing the exchange component, i.e., switching from B3LYP to BLYP, led to a significant increase in MAE, underscoring the sensitivity of dye UV–vis spectra to the exchange term. This highlights the importance of carefully selecting the exchange–correlation functional to obtain accurate predictions.

Additionally, the computed absorption wavelengths across the solvation models reveal a consistent correlation between B3LYP and B3PW91, as illustrated in Figure 2, which compares the spectra obtained using implicit solvation. This similarity suggests that, within this class of hybrid functionals, the choice between B3LYP and B3PW91 has a minimal impact on overall accuracy.

**Figure 2:**
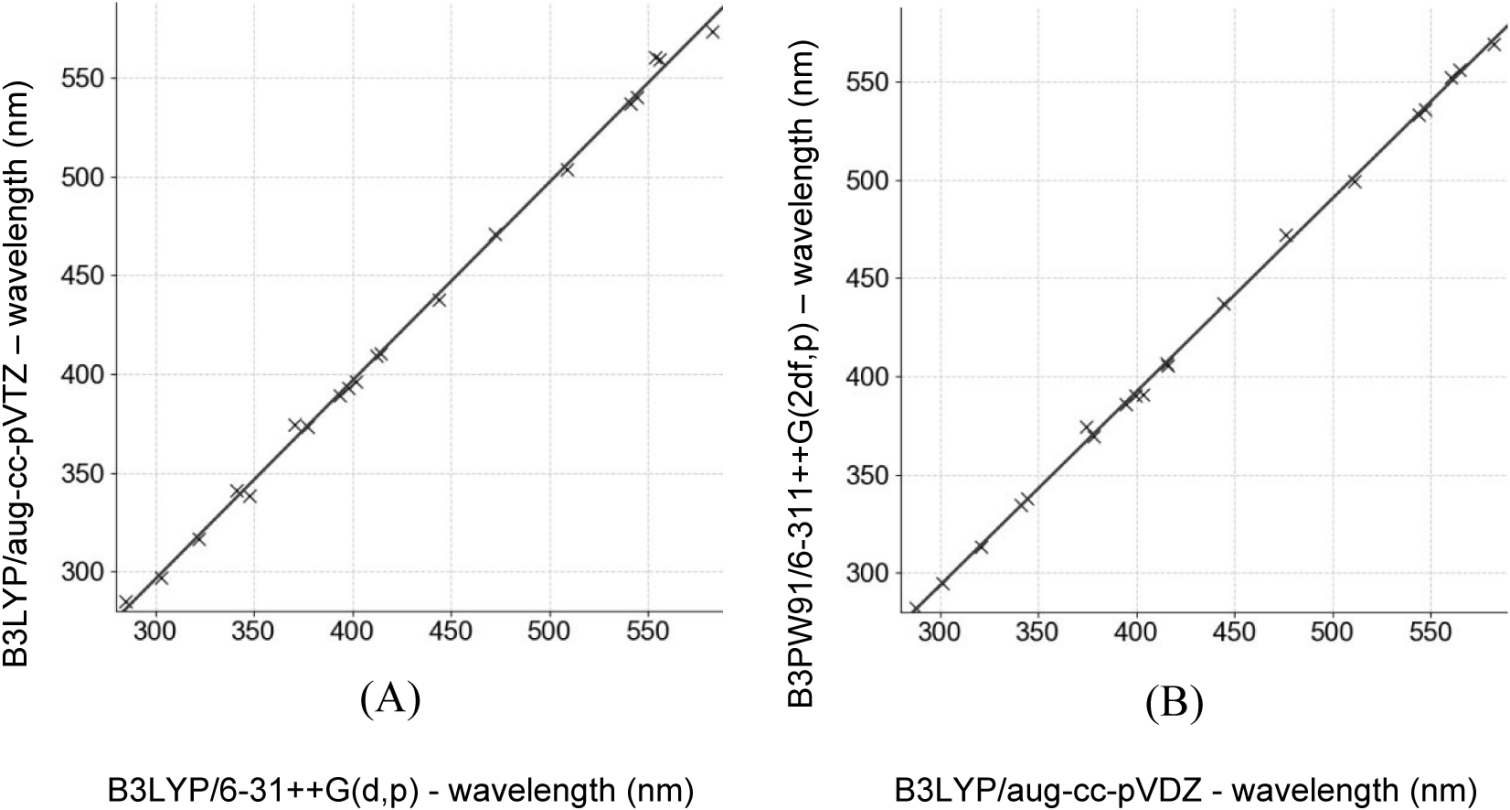
Linear regression plots comparing the calculated absorption maxima (in nanometers) for different combinations of functionals and basis sets under implicit solvation, calculated with different combinations of functionals and basis sets under implicit solvation. Regressions are shown within the plots. (A) Comparison between B3LYP/aug-cc-pVTZ and B3LYP/6-31++G­(d,p) (slope = 1.0066, intercept = −6.0011, *R* ^2^ = 0.9981); (B) comparison between B3PW91/6-311++G­(2df,p) and B3LYP/aug-cc-pVDZ (slope = 0.9863, intercept = −2.4053, *R* ^2^ = 0.9992).

The plots in Figure 2 further demonstrate that differences between B3LYP and B3PW91 are small and that the choice of the basis set does not significantly alter the computed electronic spectra. Regardless of the specific B3LYP or B3PW91 combination with the tested basis sets, the observed trends remain consistent. This finding supports the use of less computationally demanding basis sets, such as 6-31++G­(d,p), as a viable alternative without significantly compromising accuracy. Such considerations are especially relevant for large-scale calculations, where computational cost is a critical factor.

The electrostatic potential map shown in Figure 3 highlights regions of negative potential (red), which correspond to the most favorable sites for interactions with the positive end of the water dipole. In practice, water molecules were placed with their hydrogen atoms directed toward these red regions and the oxygen atoms oriented away from them, generating hydrogen-bond patterns consistent with the local electrostatic environment. The resulting microsolvated structures were then used to evaluate how such specific solute–solvent interactions influence the calculated electronic spectra. Figure 4 illustrates the effects of adding explicit water molecules to the calculated electronic spectra, highlighting how these interactions affect the absorption wavelengths.

**Figure 3:**
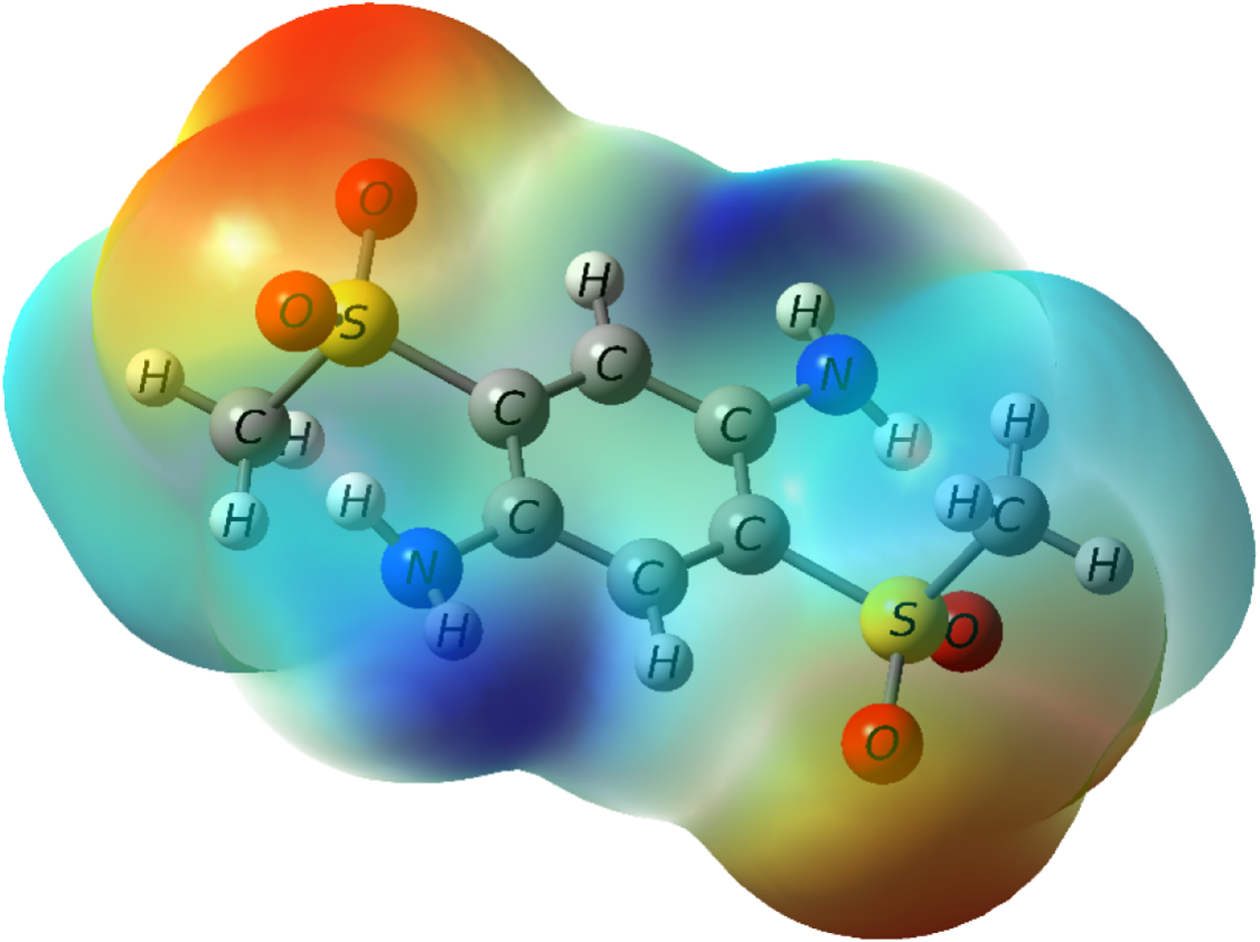
Electrostatic potential map of the molecule 2,5-bis­(methylsulfonyl)­benzene-1,4-diamine (BMESPA), calculated at the B3LYP/6-31++G­(d,p) level. Red regions indicate areas of negative electrostatic potential, while blue regions correspond to regions of positive potential.

**Figure 4:**
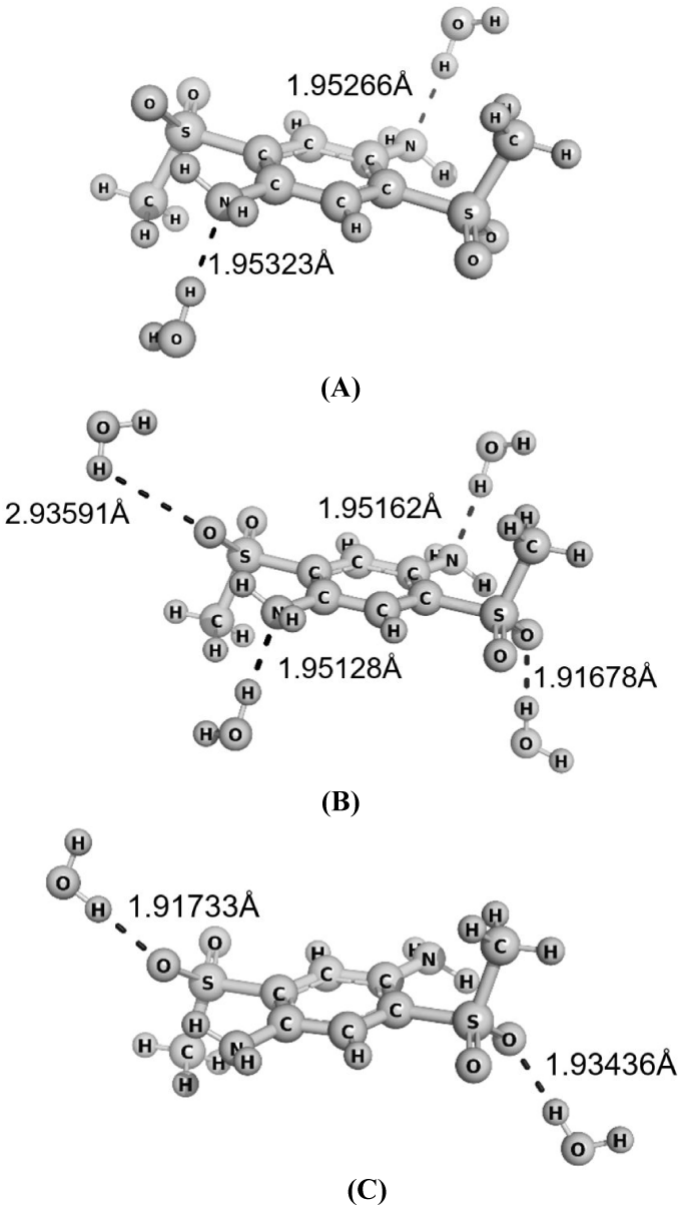
Optimized structures of the BMESPA molecule with different placements of explicit water molecules, calculated at the B3LYP/6-31++G­(d,p) level. Configuration (A): water near the NH group; (B): water near both the NH group and a region of negative electrostatic potential; (C): water placed solely in a region of negative electrostatic potential. The corresponding calculated absorption maxima are 345.74, 347.71, and 372.39 nm, respectively, while the experimental value is 377 nm.^67^

Analysis of these results reveals that placing water near regions of negative electrostatic potential (C) yielded the absorption maxima closest to the experimental values. This behavior suggests that electrostatic interactions play a key role in stabilizing the excited states, thereby reducing the discrepancy between the theoretical and experimental wavelengths. Table 2 presents data for a selected set of molecules for which explicit solvation strategies can be consistently applied and compared. All dyes, however, were included in the TD-DFT calculations and the MAE analysis reported in Table 1.

**Table 2:** Deviations (in nm) between Calculated and Experimental Electronic Absorption Maxima for a Subset of Dyes Obtained at the B3LYP/6-31++G­(d,p) Level Using Different Solvation Models: (i) Implicit Solvation; (ii) Implicit Solvation Combined with Explicit Water Molecules Placed Near OH, NH, and F Groups; and (iii) Implicit Solvation Combined with Explicit Water Molecules Placed According to Electrostatic Potential Maps^b^

		Implicit + Explicit		
Label	Implicit Solvent	HO, NH, F	Electrostatic Potential	Experimental
H_2_QZ	–12.36	–16.92	–12.7	460
HQZ	–3.6	1.95	6.52	550
HARF	–50.95	–48.24	–39.02	490
H_2_CZ	–12.09	–23.48	–28.53	420
HCZ	–34.32	–20.7	–27.95	510
H_2_AFV	–14.65	–19.55	–15.66	270
HAFV	–11.05	–8.45	–7.95	330
BMESPA	–6.60	31.26	4.61	377
C440	43.02	12.41	–3.49	342
C500	–15.89	1.06	–19.22	386
C519	11.45	11.98	11.61	426
H_2_DHBQ	–19.39	–26.56	–22.52	283
**MAE** ^a^	**20.56**	**18.55**	**16.64**	

a Mean absolute error.

b The values in the first three columns correspond to the differences in experimental and calculated values (in nm), while the last column lists the experimental absorption maxima (in nm).

The individual data in Table 2 indicate that the impact of explicit water molecules varies depending on the molecule and the solvation approach. For some cases, explicit solvation guided by the electrostatic potential led to better agreement with experimental data, whereas in others, placing water near OH, NH, or F groups increased the deviation. This variability suggests that the effectiveness of explicit solvation is not uniform and depends on the electronic structure of each molecule and the surrounding electrostatic environment. Nonetheless, overall MAE analysis indicates that the most consistent and accurate results were obtained when water molecules were placed according to electrostatic potential, supporting this method as a more reliable strategy for reproducing experimental trends.

Although more sophisticated hybrid approaches, such as QM/MM, can offer a more detailed description of specific solute–solvent interactions, they come at a substantially higher computational cost. The strategy employed in this study, explicit microsolvation guided by electrostatic potential mapping, represents a more computationally feasible alternative. Conceptually, it aligns with the microsolvation approach proposed by Pliego,^66^ offering a simplified yet chemically meaningful framework for capturing key solvent effects.

## 4 Conclusions

The comparison of exchange functionals (B vs B3) and correlation functionals (LYP vs PW91), in combination with different basis sets and solvation models, highlights the critical role of solvent effects in the TD-DFT calculations of UV–vis spectra. Among the approaches tested, B3LYP and B3PW91 provided consistent and accurate results, confirming their suitability for describing the electronic structure of coumarin- and anthraquinone-based dyes. The substitution of the B exchange functional with B3 significantly improved spectral predictions, whereas variations between the LYP and PW91 correlation functionals produced only minor differences. Thus, for π-conjugated organic dyes closely related to the coumarin and anthraquinone classes investigated here, hybrid functionals based on the B3 exchange component are found to offer a good compromise between accuracy and computational efficiency within the present benchmark.

Basis set analysis showed consistent performance across all systems, with simpler sets such as 6-31++G­(d,p) yielding results comparable to those of larger and more computationally demanding alternatives. This finding supports the use of lower-cost basis sets in large-scale studies without a significant loss of accuracy.

Notably, the use of electrostatic potential maps to guide the placement of explicit water molecules resulted in improved agreement with the experimental spectra, producing the lowest mean absolute errors among the tested solvation strategies. Although the effectiveness of this approach varies with the molecular structure, it offers a computationally efficient and physically motivated alternative to more demanding hybrid methods such as QM/MM. Further expansion of the molecular data set may help clarify the specific conditions under which this ESP-guided microsolvation strategy is most effective.

Finally, it is emphasized that the present study is deliberately limited to vertical absorption maxima (λ_max_) and associated error metrics and does not attempt to reproduce full spectral profiles. Combining the ESP-guided microsolvation protocol with more elaborate treatments of vibronic structure and line broadening for selected dyes is envisaged as the focus of future, complementary work.

## Supplementary Material



## Data Availability

Molecular charge, multiplicity, optimized geometries, calculation levels, basis sets, and excitation details are provided in the Supporting Information file in a machine-readable text format. All quantum chemical calculations were performed with the commercial software Gaussian 09. Output files generated during the calculations are available from the authors upon reasonable request. These files are not required to reproduce the essential results or to illustrate the reported methodology since all necessary data for validation are contained in the Supporting Information. Graphs and figures were generated using Python (matplotlib library, open source) and ChemSketch (freeware).

## Notes

Optimized geometries and TD-DFT excitation data; for each case, the charge and multiplicity of the molecule or ion are reported above the optimized Cartesian coordinates (in Å); the selected electronic transition, chosen for its proximity to the experimental absorption maximum and, when applicable, its higher oscillator strength, is presented in the standard Gaussian output format (TXT)
